# Clinical characteristics associated with sustained ventricular arrhythmia among acute myocarditis patients: a multicenter case-control study

**DOI:** 10.1007/s10840-026-02408-5

**Published:** 2026-08-04

**Authors:** Moshe Rav-Acha, Daniel Charles Pipilas, Esra Gumuser, Arik Wolak, Tomer Ziv-Baran, Yoav Michowitz, Elad Asher, Itshak Amsalem, Tal Hasin, Michael Glikson, Eden Sinai, Chee Yuan Ng

**Affiliations:** 1 Jesselson Integrated Heart Center and Eisenberg R&D Authority, Shaare Zedek Medical Center, Hebrew University, Jerusalem, Israel; 2 Cardiac Arrhythmia Center, Massachusetts General Hospital, Harvard University, Boston, MA USA; 3 Gray Faculty of Medical & Health Sciences, Tel-Aviv University School of Public Health, Tel-Aviv University, Tel-Aviv, Israel

**Keywords:** Acute myocarditis, Ventricular arrhythmia

## Abstract

**Background:**

Clinical features associated with sustained ventricular arrhythmias (sVA) during acute myocarditis (AM) are poorly defined.

**Objective:**

Identify simple clinical characteristics of arrhythmogenic AM patients who develop sVA during acute-phase hospitalization.

**Methods:**

Case-control study of AM patients hospitalized in two tertiary centers from 2000 to 2020. Patients with documented sVA during AM hospitalization (cases) were compared with AM patients without VA (controls). Patients with history of myocardial infarction, heart failure or VA were excluded. Univariate and multivariable analyses were used to evaluate clinical parameters associated with occurrence of sVA during AM hospitalization.

**Results:**

Study included 41 cases with sVA and 137 controls without any VA during AM hospitalization period. VAs included monomorphic VT and polymorphic VT/VF in 24/41 (58.5%) and 17/41 (41.5%) cases, respectively. Older age, syncope, palpitations, hemodynamic instability, intraventricular conduction delay on ECG, and reduced LV function (LVF) were associated with increased sVA occurrence on univariate analysis. Multivariable model including age, gender, admission ECG, and LVF revealed age (OR 1.027/year, 95% CI [1.015, 1.052]; *p* = 0.038), intraventricular conduction delay (OR 5.55, 95% CI [2.237, 8.346]; *p* < 0.001), and reduced LVF (OR 4.38, 95% CI [1.783, 9.081]; *p* = 0.002) to be independently associated with sVA. Applying these characteristics, we could delineate AM patients with differing prevalence of sVA during hospitalization.

**Conclusions:**

Few clinical parameters, readily available upon admission, including older age, intraventricular conduction delay, and reduced LVF, are associated with acute-phase sVA in AM. Once validated by larger studies, these parameters may assist decision-making regarding level of care and ECG monitoring.

**Graphical Abstract:**

**Figure:**
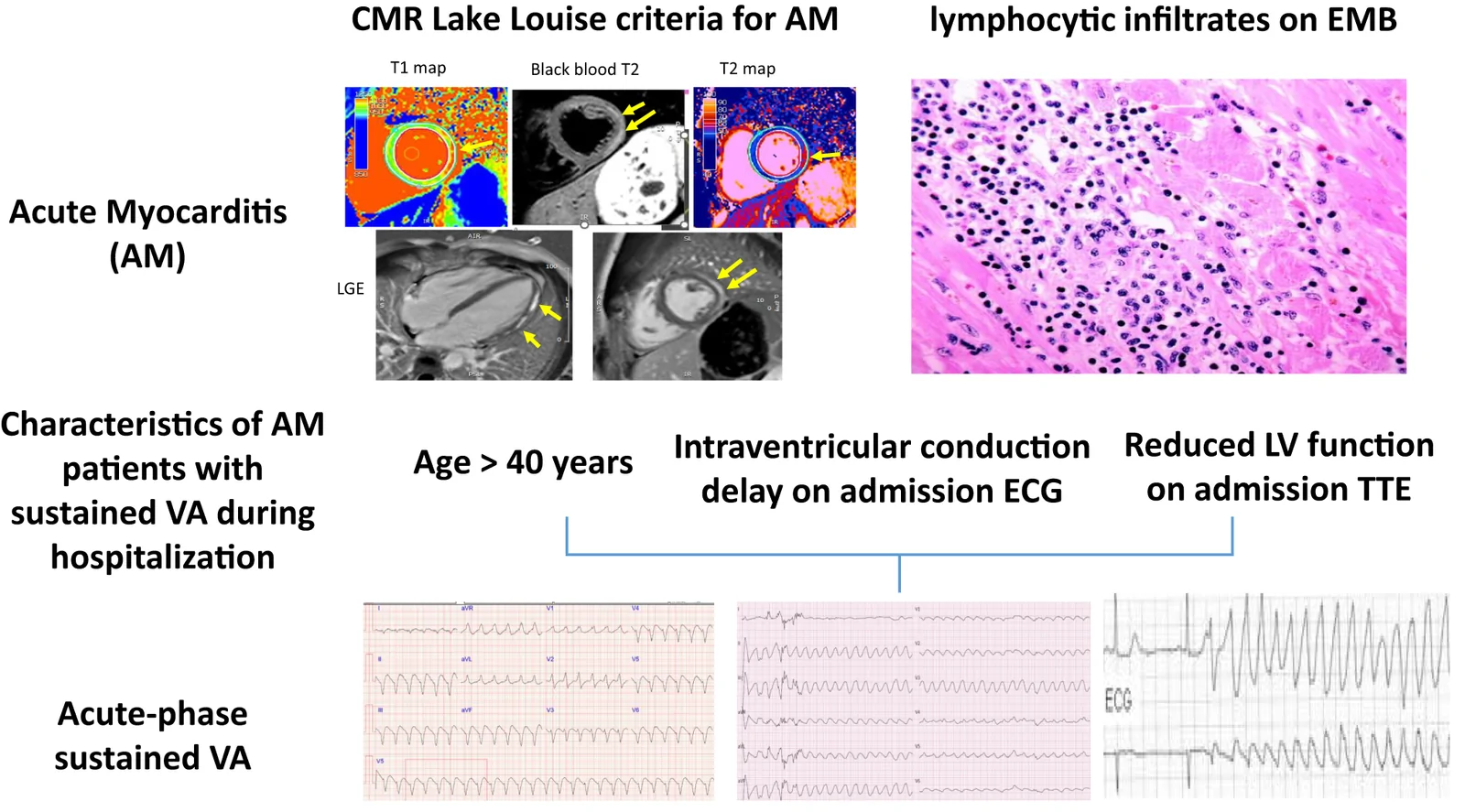

**Supplementary Information:**

The online version contains supplementary material available at https://doi.org/10.1007/s10840-026-02408-5.

## Introduction

Acute myocarditis (AM) has a wide range of clinical presentations, evolutions and prognoses, ranging from a subclinical disease with complete recovery and excellent long-term prognosis to fulminant progressing heart failure with malignant ventricular arrhythmias (VA) [1–5]. A quarter of AM patients are suggested to have arrhythmias, a third of which (~ 8%) being VA [6]. Specifically for VF or cardiac arrest, these were reported in 2.5% of AM cases [1, 5]. Although AM patients are known to have an elevated long-term risk of sVA [1–3, 6, 7], data regarding clinical characteristics associated with sVA during the acute-phase illness is limited [8–10].

To date, hospital admission and continuous ECG monitoring are recommended in high-risk AM patients presenting with sVA, high-degree atrio-ventricular block, acute heart failure, cardiogenic shock, and New-York Heart Association (NYHA) III-IV symptoms [1, 3, 5, 8]. However, no clear recommendation is given for the majority of AM patients who do not present with the above high-risk features, but may still develop VA during hospitalization. This might be related to the absence of clinical characteristics of AM patients who are at risk for acute-phase sVA. Thus, in clinical practice, many AM patients are admitted to internal medicine wards without continuous monitoring and are discharged within a few days. Evaluation of clinical parameters associated with acute-phase sVA is crucial in order to identify risk subgroups for which hospitalization in the cardiology ward or intensive care unit (ICU) with continuous ECG monitoring may be warranted. Accordingly, our study aim was to compare the clinical characteristics of AM patients with and without VA during AM hospitalization in order to identify simple clinical parameters, readily available upon admission, which are associated with *acute-phase sVA*.

## Methods

A retrospective case-control study of patients hospitalized for AM in the cardiology ward or intensive cardiovascular care unit (ICCU) between 2000–2020, in two large tertiary health centers, Shaare Zedek Medical Center, Jerusalem, Israel, and Mass General Brigham, Boston, MA, USA. In our study, AM patients with documented sVA during their acute-phase hospitalization (defined as ‘cases’) were compared with AM patients with no VA during hospitalization (defined as ‘controls’). Notably, consecutive AM patients admitted with sVA during acute-phase hospitalization, fulfilling the inclusion and exclusion criteria (listed below), were included. A simple randomization and selection process was employed. For each ‘case’ with sVA, we aimed to select four consecutive acute-phase myocarditis patients without VA from the same medical center and year, as controls. These controls were selected based on chronological proximity: Selecting the two patients hospitalized immediately prior to the case and the two patients hospitalized immediately following the case. In cases with < 4 controls available, all were included. Although controls were unmatched to cases for clinical characteristics, they were selected based on chronological proximity and hospitalization center as the cases.

Cases were identified via computerized search for both AM diagnosis and VA during the acute-phase hospitalization (‘index’ hospitalization). Thereafter, patient charts were thoroughly reviewed by a senior cardiologist to verify AM diagnosis as well as the presence and type of VA.

Both VA cases and controls had a confirmed diagnosis of AM. Diagnosis of AM was based on the combination of suggestive clinical symptoms, elevated myocardial injury and inflammatory markers, absence of obstructive (> 50%) coronary lesion on coronary angiography or coronary CT angiogram (CCTA), and confirmed using Lake Louise criteria (LLC) on cardiac magnetic resonance (CMR) [11, 12] and/or endomyocardial biopsy (EMB), in accordance with the myocarditis consensus documents [1–3, 5, 8].

Inclusion criteria:

1. Confirmed AM diagnosis as detailed above; symptom onset within 30 days prior to hospitalization [2, 5], with no clinical evidence of prior myocarditis.
2. VA cases with a documented sustained monomorphic, polymorphic VT, or VF (lasting ≥ 30 seconds or necessitating cardioversion/defibrillation) during the AM index hospitalization. Documentation of the VA was achieved via continuous ECG monitoring or 24-h ECG holter during hospitalization.

Exclusion criteria:

1. Obstructive coronary artery disease (> 50% lesion) on cardiac catheterization or CCTA.
2. History of myocardial infarction (MI), prior sVA/cardiopulmonary resuscitation, or prior myocarditis.
3. History of cardiomyopathy, LV dysfunction on TTE, or congestive heart failure symptoms prior to current admission.
4. Family history of cardiomyopathy, sudden cardiac death (SCD), or channelopathy.

### Clinical parameters evaluated

In order to evaluate clinical characteristics of AM patients with *in-hospital sVA*, we compared AM cases with sVA and AM controls without VA, evaluating the association between various clinical parameters, present on admission, and occurrence of sVA during hospitalization. These parameters included: (a) patient demographic data, background illness, and presenting symptoms; (b) admission ECG data, including QRS width (during sinus rhythm), and presence of intraventricular conduction delay as defined by the AHA/ACCF/HRS consensus on intraventricular conduction disturbance on ECG [13]; (c) laboratory inflammatory markers including WBC, CRP and myocardial injury markers such as Troponin and CPK; (d) trans-thoracic echocardiography (TTE) data upon admission including LV function, LV end-diastolic diameter, and presence of pericardial effusion; (e) CMR data acquired during index hospitalization including the presence and dominant location of late gadolinium enhancement (LGE).

Intraventricular conduction delay in our study included LBBB, RBBB, and non-specific intraventricular conduction disturbance, in accordance with the AHA/ACC recommendation for standardization and interpretation of ECG [13].

Left ventricular function (LVF) on admission was categorized as preserved or reduced according to LV ejection fraction of ≥ 50% or < 50% on TTE, respectively. Assessment of LVEF was performed via modified biplane Simpson Method. Moderate-severe and severe LV dysfunction were defined by LVEF < 35%.

### Statistical analysis

Categorical variables were summarized as frequency and percentage. Continuous variables were evaluated for normal distribution using histogram and Q-Q plot, and reported as mean ± SD or median and 25%, 75% inter-quartile range (IQR), as appropriate. Chi-square and Fisher exact tests were used to study the association between categorical variables and sVA occurrence, while the association between continuous variables and sVA occurrence was done via T-test and Mann-Whitney Test for variables with normal and non-normal distribution, respectively. Parameters which were significantly associated with the sVA occurrence on univariate analysis were considered thereafter for multivariable analysis to evaluate their independent association with the study endpoint of sVA occurrence during acute-phase myocarditis. Due to the relatively small number of cases with sVA (*n*= 41) leading to limited number of parameters in a multivariable model [14], we evaluated two different models, to evaluate a few combinations of clinically relevant parameters,

Receiver operating characteristic (ROC) analysis was then performed using the probabilities derived from the multivariable logistic regression models to evaluate the discrimination ability of the regression models.

Youden J index, which is a statistical method to determine the optimal cutoff point for a binary classification, was used to identify the optimal age cutoff value to best discriminate between the presence or absence of acute-phase sVA.

All statistical tests were two-tailed, and *p* < 0.05 was considered statistically significant. SPSS software was used for all statistical analyses (IBM SPSS Statistics, version 27, IBM Corp, Armonk, New York, USA, 2020). The sample size calculation is shown in the Supplement Appendix. The study was approved by the ethical committee in both participating medical centers. Noteworthy, study cases and controls were all hospitalized prior to the COVID-19 pandemic in 2020. Thus, our study does not include COVID-19-related myocarditis.

## Results

**Fig. 1:**
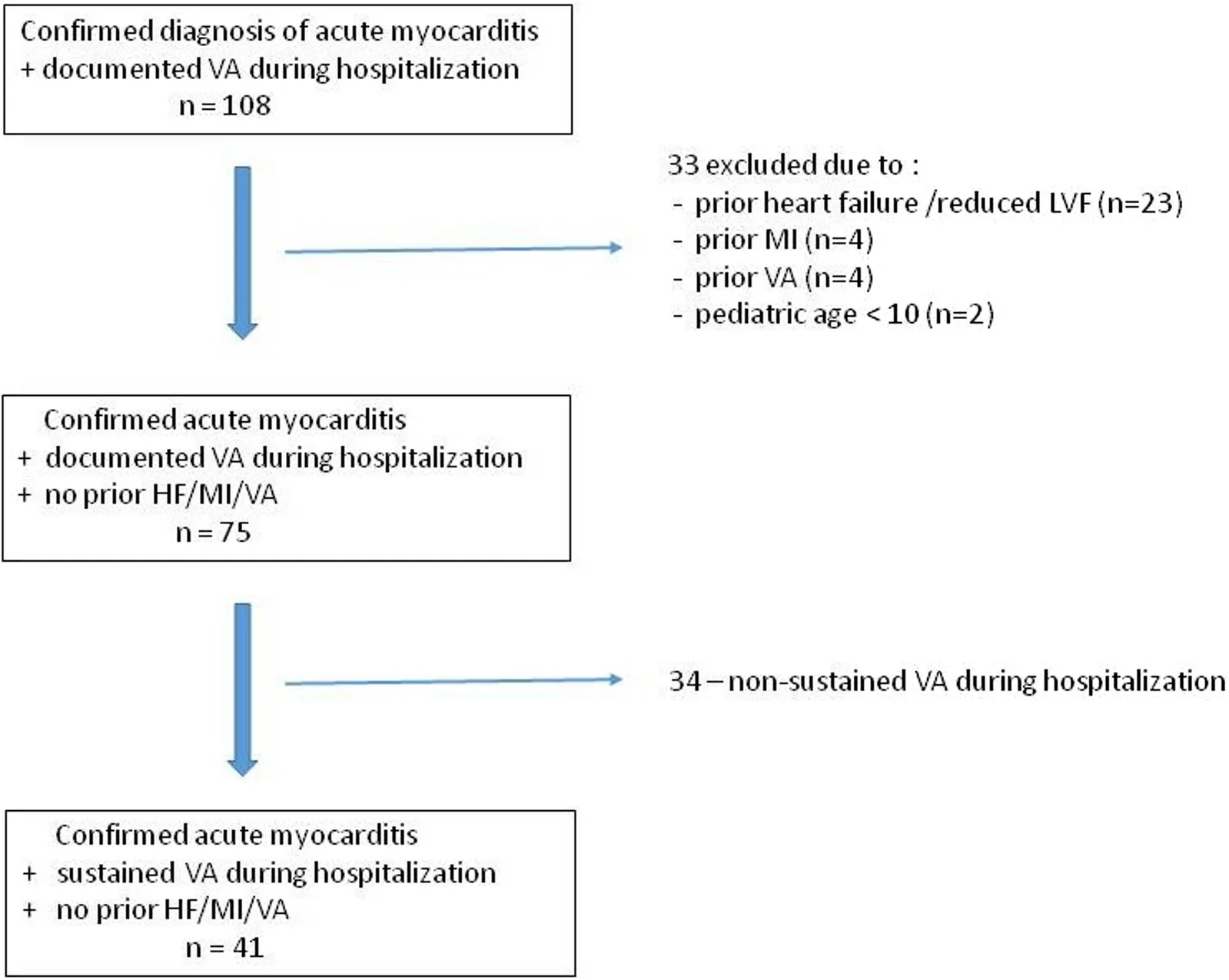
Cases inclusion and exclusion flow-chart

A total 0f 108 cases with confirmed AM and documented VA during their acute-phase hospitalization were identified. After excluding patients with prior MI, prior LV dysfunction, prior VA, and those with non-sustained VA, our study included 41 AM cases with sustained VA (sVA) during their index hospitalization (Fig. 1). For each case of AM with sVA, 3–4 controls with confirmed AM and without VA were randomly selected. Thus, our study included 41 sVA cases and 137 controls without VA who were admitted with AM diagnosis. Sustained VA during index hospitalization included sustained Monomorphic VT (sMMVT), sustained Polymorphic VT (sPMVT), and VF in 24/41 (58.5%), 6/41 (14.6%), and 11/41 (26.9%) cases, respectively. The sVA occurred at 19 [3, 68] hours after admission, and the overall hospitalization duration was 6 [3, 11] days.

### Patients’ background, admission characteristics, and CMR/EMB results

Medical background, presenting symptoms, admission labs, ECG, and TTE results, as well as CMR data of both cases and controls, are shown in Table 1. All patients had confirmed AM diagnosis, via CMR, EMB, or both. CMR was performed in 147 patients, all meeting ≥ 2/3 Lake Louise criteria. An EMB was performed in 49 patients, revealing lymphocytic myocarditis, giant-cell myocarditis, eosinophilic myocarditis, and granulomatous myocarditis in 26, 12, 9, and 2 cases, respectively.

**Table 1:** Comparison of patient’s characteristics between cases with sustained VA and controls without VA during acute phase myocarditis

*Parameter*	*Total* (*n* = 178)	*VA* (*n* = 41)	*No VA* (*n* = 137)	*P*
Age (years)	40 [27,53]	48 [40, 56]	36 [25, 51]	**< 0.001**
Female	58 (32.6%)	18 (43.9%)	40 (29.2%)	0.077
Medical background				
Hypertension	33 (18.5%)	9 (21.9%)	24 (17.5%)	0.521
Diabetes mellitus	8 (4.5%)	4 (9.7%)	4 (2.9%)	0.167
Chronic renal failure*	3 (1.7%)	2 (4.9%)	1 (0.7%)	0.266
Smoking	35 (19.6%)	6 (14.6%)	29 (21.1%)	0.355
Alcohol consumption**	22 (12.3%)	6 (14.6%)	16 (11.7%)	0.614
Presenting symptoms				
Fever	46 (25.8%)	9 (21.9%)	37 (27%)	0.516
Chest pain	119 (66.8%)	12 (29.2%)	107 (78.1%)	**< 0.001**
Dyspnea	76 (42.7%)	20 (48.8%)	56 (40.9%)	0.369
Syncope	14 (7.8%)	7 (17%)	7 (5.1%)	**0.004**
Palpitations	15 (8.4%)	9 (21.9%)	6 (4.4%)	**0.002**
Hemodynamic instability	34 (19.1%)	21 (51.2%)	13 (9.5%)	**< 0.001**
Lab				
WBC (Normal: 4–10^^3^/L)	9 [7,11.4]	9.7 [8, 13.7]	8.9 [7.3, 10.6]	0.055
CRP (Normal: 0–5 mG/L)	33 [7, 98]	48 [22, 99]	31 [6, 90]	0.63
Elevated Troponin	144/166 (86.7%)	29/37 (78.4%)	115/129 (89.1%)	0.161
CK admission (Normal: 40–150 IU/L)	327 [125, 620]	287 [119, 968]	342 [131, 587]	0.720
LDH (Normal: 120–220 IU/L)	474 [313, 690]	462 [310, 919]	475 [318, 678]	0.350
ECG (*n* = 175) on admission				
Intraventricular conduction delay	31/175 (17.7%)	17/41 (41.5%)	14/134 (10.4%)	**< 0.001**
RBBB	19/175 (10.8%)	13/41 (31.7%)	6/134 (4.5%)	**< 0.001**
LBBB	6/175 (3.4%)	3/41 (7.3%)	3/134 (2.2%)	0.283
Non-specific IVCD***	6/175 (3.4%)	1/41 (2.5%)	5/134 (3.7%)	0.766
QRS width admission (ms)	92 [84,102]	105 [88, 129]	90 [83, 98]	**0.001**
ST-elevation on admission (n, %)	65/175 (37.1%)	13/41 (31.7%)	52/134 (38.8%)	0.410
TTE (*n* = 169) on admission				
Reduced LV function (LVEF < 50%)	84/169 (49.7%)	32/41 (78%)	52/128 (40.6%)	**< 0.001**
Moderate/severe or severe LV dysfunction (LVEF < 35%)	38/169 (22.5%)	16/41 (39%)	22/128 (17.2%)	**0.003**
LVEDD on admission (mm)	49 [45,54]	49 [44, 55.5]	49.5 [45, 54]	0.7
Pericardial effusion on admission	33/169 (19.5%)	16/41 (39%)	17/128 (13.3%)	**0.002**
CMR (*n* = 147)				
LGE any	140/147 (95.3%)	26/28 (92.8%)	114/119 (95.7%)	0.780
LGE anteroseptal	42/147 (28.5%)	11/28 (39.3%)	31/119 (26%)	0.240
LGE inferolateral	98/147 (66.7%)	15/28 (53.5%)	83/119 (69.7%)	0.102
Abnormal T1 weighted sequence	75/147 (51%)	12/28 (42.8%)	63/119 (52.9%)	0.35
Abnormal T2 weighted sequence	124/147 (84.3%)	20/28 (71.4%)	104/119 (87.4%)	0.063
Medications during hospitalization				
Beta adrenergic blockers	110 (61.8%)	30 (73.1%)	80 (58.4%)	0.07
ACEi or ARB	120 (67.4%)	33 (80.5%)	87 (63.5%)	**0.04**
MRA	40 (22.5%)	13 (31.7%)	27 (19.7%)	0.1
Amiodarone	1 (0.6%)	0 (0%)	1 (0.7%)	0.8

* Defined by Creatinine > 1.4 mg%; ** Considered if > 5 glasses/week; *** IVCD = Intra-Ventricular Conduction Delay Categorical variables presented as n (%) and continuous as median [IQR]

**Table 2:** Multivariable analyses for sVA during AM hospitalization, based on two combinations of admission clinical parameters, associated with sVA on univariate analysis

A	*P*	OR	95.0% CI for Exp(B)	
			Lower	Upper
Age	**0.038**	1.027	1.015	1.052
Gender (female)	0.161	1.870	0.814	4.343
Intraventricular conduction delay on ECG	**< 0.001**	5.551	2.237	8.346
Reduced LV function (EF < 50%) compared with Preserved LV function (EF ≥ 50%)	**0.002**	4.385	1.783	9.081
B	*P*	OR	95.0% CI for Exp(B)	
			Lower	Upper
Age	0.366	1.013	0.991	1.042
Chest pain	**< 0.001**	0.145	0.070	0.412
Syncope/palpitations	**0.003**	4.812	1.872	9.850
Intraventricular conduction delay on ECG	**0.001**	5.963	2.323	11.831
Reduced LV function (EF < 50%) compared with Preserved LV function (EF ≥ 50%)	**0.029**	3.239	1.615	8.357

### Clinical characteristics associated with acute-phase sVA on univariate analysis

A univariate analysis comparing patient characteristics, presenting symptoms, admission lab results, ECG, and imaging data between cases and controls is shown in Table 1. Background illness was similar among cases and controls. VA cases were significantly older than controls (48 [40, 56] vs. 36 [25, 51] years; *p* < 0.001).There were more cases presenting with syncope and palpitations compared with controls (17% vs. 5.1%; *p* = 0.004; and 21.9% vs. 4.4%; *p* = 0.002, respectively), and less cases presenting with chest pain (29.2% vs. 78.1%; *p* < 0.001). More cases were hemodynamically unstable on presentation (51.2% vs. 9.5%; *p* < 0.001). Admission ECG showed more cases with intraventricular conduction delay, compared with controls (41.5% vs. 10.4%; *p* < 0.001).

Admission TTE demonstrated a higher proportion of cases with reduced LVF (EF < 50%), including an increased prevalence of moderate-to-severe or severe LV dysfunction (EF < 35%) among cases as compared with controls (78% vs. 40.6%; *p* < 0.001; and 39% vs. 17.2%; *p* = 0.003, respectively; Table 1).

Our aim was to identify *simple* clinical parameters *available upon admission*, which are independently and significantly associated with in-hospital sVA. Thus, we did not include CMR nor EMB data in the multivariate analysis, as these ‘advanced’ examinations were performed relatively late during hospitalization, and their results were thus unknown upon admission.

### Clinical parameters associated with acute-phase sVA by multivariable analysis

Multivariable logistic regression analysis for acute-phase sVA, incorporating parameters significantly associated with sVA in the univariate analysis, including age, gender, intraventricular conduction delay on admission ECG, and reduced LVF on admission TTE, revealed that age (OR 1.027/year, 95% CI [1.015, 1.052]; *p* = 0.038), intraventricular conduction delay (OR 5.551, 95% CI [2.237, 8.346]; *p* < 0.001), and reduced LVF (OR 4.385, 95% CI [1.783, 9.081]; *p* = 0.002) were all significantly and independently associated with acute-phase sVA (Table 2A).

A second multivariable analysis for sVA including age, chest pain, syncope/palpitations, intraventricular conduction delay on admission ECG, and reduced LVF revealed chest pain (OR 0.145, 95% CI [0.07, 0.412; *p* < 0.001) to be significantly associated with reduced sVA, while syncope/palpitations (OR 4.812, 95% CI [1.872, 9.850]; *p* = 0.003), intraventricular conduction delay (OR 5.963, 95% CI [2.323, 11.831]; *p* = 0.001), and reduced LVF (OR 3.239, 95% CI [1.615, 8.357]; *p* = 0.029) were associated with increased acute-phase sVA (Table 2B).

ROC analysis using the probabilities derived from the above multivariate logistic regression models, suggested a good predictive ability for the study endpoint of sVA during AM hospitalization in model 2 A (AUC 0.82, 95% CI [0.74, 0.88]; *p* < 0.001), and a better predictive ability in model 2B (AUC 0.85, 95% CI [0.82, 0.92]; *p* < 0.001) (Supp Fig. 1).

**Fig. 2:**
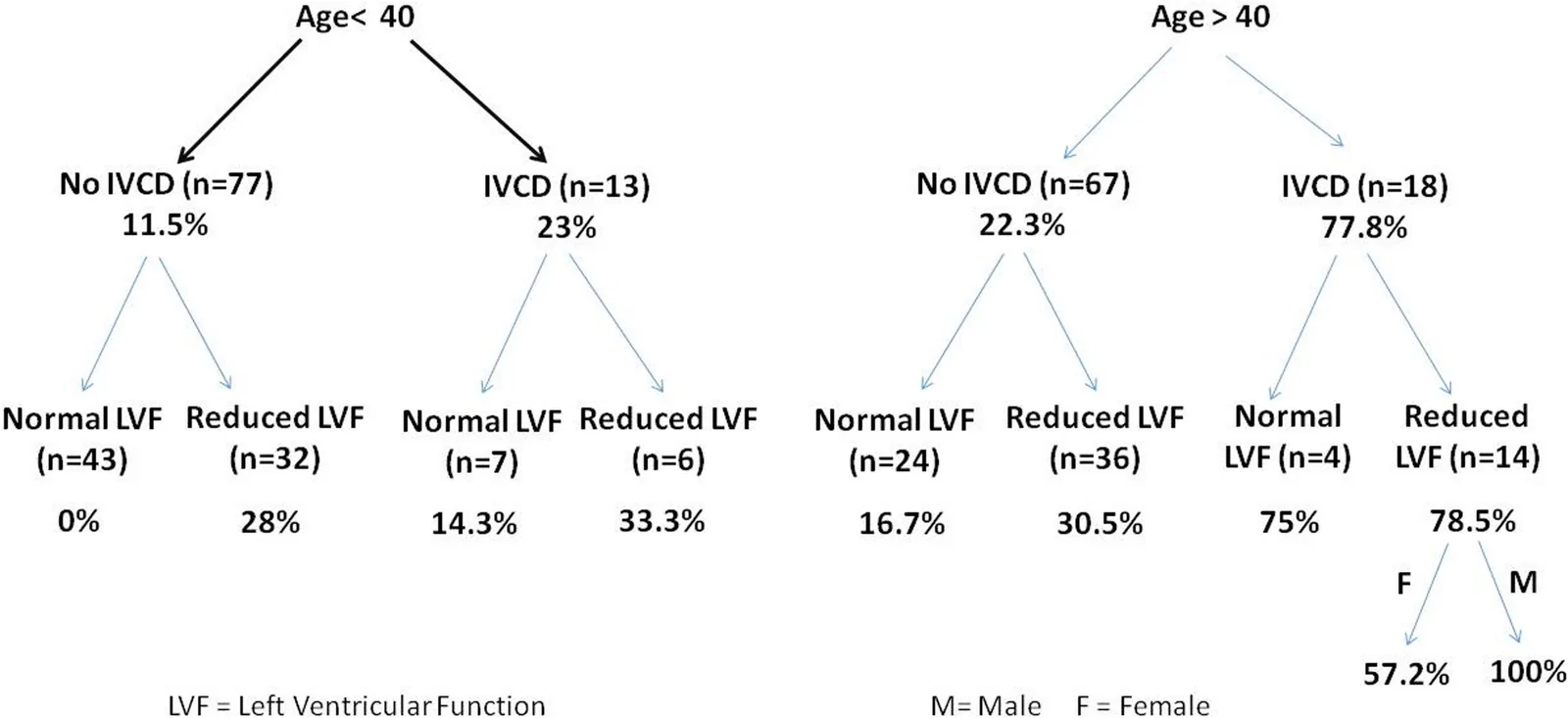
Sustained VA prevalence in our study patients according to main clinical characteristics associated with acute-phase sVA, namely older age, intraventricular conduction delay, and reduced LV function, delineating patients with low and high prevalence of sVA during AM hospitalization

A diagram describing the prevalence of acute-phase sVA in our study according to the main clinical parameters associated with sVA, namely age, intraventricular conduction delay on admission ECG, and reduced LVF, is shown in Fig. 2. Using the Youden J index, age > or ≤ 40 years was found as the optimal age cutoff to best discriminate between cases with sVA and controls without VA (yielding a maximal index of 0.3450). Notably, younger AM patients (≤ 40 years) with normal intraventricular conduction on ECG and preserved LVF had no sVA, while older patients (> 40 years) with intraventricular conduction delay on ECG and reduced LVF had a prevalence of 78% sVA during hospitalization (Fig. 2). The prevalence of acute-phase sVA according to the various combinations of these three clinical parameters is presented in Fig. 2.

## Discussion

In this multicenter case-control study including 41 AM cases with sVA and 137 AM controls without VA during index hospitalization, we found multiple clinical parameters, present upon admission, to be associated with in-hospital sVA. Characteristically, AM cases were older (> 40 years), had increased tendency to present with syncope or palpitations rather than chest pain, had increased prevalence of intraventricular conduction delay on ECG, and reduced LVF on admission TTE. In multivariable analyses, older age, syncope/palpitations, intraventricular conduction delay, and reduced LVF were significantly associated with increased prevalence of sVA, while chest pain was associated with reduced sVA prevalence. Using these clinical characteristics, we could delineate subgroups with differing prevalence of in-hospital sVA, among the study patients. A sub-analysis of hemodynamically stable AM patients (shown in the Supplementary Appendix) revealed that the main clinical parameters associated with sVA among the whole study population, namely older age, intraventricular conduction delay, and reduced LVF, were significantly associated with sVA among hemodynamically stable AM patients as well.

Many of the VA cases in our study were included in our group’s prior publication, evaluating predictors for *long-term*sVA recurrence in AM patients with acute-phase VA [7]. This prior study enrolled 69 AM patients with documented acute-phase VA (both sustained and non-sustained) and assessed their long-term risk of recurrent sVA and/or death. Over a median follow-up (F/U) period of 5.5 years, 27/69 (39%) patients reached the composite endpoint including recurrent sVA and/or death. Two possible predictors for long-term sVA recurrence and death were identified: VA type during the acute-phase hospitalization (specifically, sustained monomorphic VT), and pre-discharge LV dysfunction. Using these predictors, AM patients were stratified into subgroups with low- and high-risk for recurrent sVA in whom an ICD might be considered [7]. A similar prevalence of recurrent sVA after AM has been shown by others, reporting 30% VA recurrence among 185 AM patients during 27 months F/U [15], and 39% VA recurrence among 28 AM patients during 36 months F/U [6]. Other studies have also implicated persistent LV dysfunction (following AM discharge), prolonged QRS duration, and the presence of mid-myocardial anteroseptal LGE on CMR as predictors of long-term VA [6, 15–21]. Importantly, the aim of our current study was to identify clinical parameters associated with *acute-phase*sVA. Unlike our prior study [7], the current study included only sustained VA cases to highlight parameters specifically related to sustained VA.

To date, there is sparse data regarding clinical characteristics of AM patients who develop sVA during their acute-phase period. In contrast with a prior seminal paper on myocarditis, suggesting that ECG findings are non-specific and are not associated with acute-phase VA [15], our study found that intraventricular conduction delay was significantly and independently associated with acute-phase VA. Notably, most prior studies evaluating ECG findings in AM patients [8, 10, 15, 18, 22] have shown that a prolonged QRS, predominantly comprising LBBB, RBBB, and non-specific intraventricular conduction delay (resembling our definition of intraventricular conduction delay), predicts a poor *long-term* prognosis, including the need for heart transplantation and cardiac death. However, these studies did not specifically evaluate its association with *acute-phase* sVA. To our knowledge, our study is the first to suggest that intraventricular conduction delay, found on AM patients’ admission ECG, is significantly associated with *acute-phase* sVA.

In our study, LV dysfunction was significantly and independently associated with sVA during AM. Although multiple prior studies show that reduced LVF predicts *long-term* mortality [2, 4, 7, 8, 10, 23, 24] and *late *VA events occurring after the acute-phase period [4, 7, 17], to the best of our knowledge only two prior studies evaluated LVF association with *in-hospital *VA events in AM [24, 25]. The first study included pediatric AM patients in whom abnormal longitudinal and segmental TTE strain were predictive of in-hospital arrhythmia, including VA [25]. The second, including adult AM patients, showed reduced LVF to be significantly associated with in-hospital VA on univariate analysis and with a combined endpoint of de-compensated heart failure and VA events, on multivariable analysis [24]. Importantly, in our study even mild LV dysfunction was significantly associated with acute-phase VA. This is in line with the Pretto study [19], which included 184 myocarditis patients with acute-phase VA (123 active myocarditis and 61 previous myocarditis patients), where mean EF on admission TTE was 49%, suggesting that a significant proportion of AM patients with acute-phase VA had preserved or mildly reduced LVF.

The older age characterizing our VA cases is consistent with prior studies showing that myocarditis patients with VA are typically of older age [16, 19, 23]. The reduced prevalence of in-hospital sVA in AM patients presenting with chest pain is in line with prior studies suggesting a favorable short- and long-term prognosis for AM presenting with chest pain [4, 8, 17]. One possible explanation for the favorable prognosis of chest pain in AM may be its association with relatively mild myocarditis, as suggested by its association with preserved LVF in our study (preserved LVF seen in 69/119 [58%] patients with chest pain compared with 11/59 [18.6%] patients without chest pain, *p* < 0.001).

We did not find a significant association between anteroseptal LGE on CMR and acute-phase sVA. This is in line with prior studies showing anterospetal LGE to predict long-term VA events after AM but not during AM hospitalization. [15–17, 20].

Finally, the association of older age, intraventricular conduction delay on ECG, and reduced LVF with acute-phase sVA in hemodynamically stable AM patients is of specific clinical relevance since these patients are often admitted to internal medicine wards without continuous ECG monitoring. If confirmed by larger studies, these simple and readily available clinical parameters may help guide patient placement in a cardiology ward with continuous ECG monitoring.

## Limitations

Our study has several limitations: (1) It is based on a retrospective analysis conducted across two medical centers over a 20-year period, during which clinical practices may have evolved and differed between centers. To mitigate this, controls were selected from the same medical center and calendar year as the cases. (2) Due to clinical constraints, a 1:4 matching ratio was not consistently achieved for all 41 cases, resulting in a final control group of 137 instead of 164. However, a post-hoc power analysis confirmed that this reduction had a negligible impact, with statistical power remaining at 79.8%, consistent with the conventional 80% threshold. (3) Only 49/178 (27.5%) patients underwent EMB, and thus we do not have histological data on majority of our patients. (4) Genetic data were not available, precluding assessment of inherited arrhythmogenic conditions (e.g., desmosomal protein mutations) known to be associated with ventricular arrhythmia. (5) We did not collect detailed hemodynamic data during ventricular arrhythmia episodes, preventing analysis of predictors of hemodynamically unstable events. Nonetheless, we propose that the occurrence of sustained ventricular arrhythmia, regardless of hemodynamic status, identifies a higher-risk subgroup that may benefit from intensified ECG monitoring during the acute phase. (6) Data on symptom duration prior to index admission were not available. Thus, we could not evaluate whether specific symptoms (such as chest pain) resulted in earlier admission, contributing to reduced AM ‘severity’ with less acute-phase sVA. (7) The relatively small number of cases with sVA limited the number of variables which could be included in multivariable analyses and precluded development of a single predictive model. Furthermore, because controls were not matched for potential clinical confounders, residual confounding may have influenced the observed associations despite multivariable adjustment and we could not rule out the possibility that variables with weak association would not be identified in our current study.

Given these limitations, our results should be considered hypothesis-generating, requiring confirmation and external validation before informing clinical decision-making or developing of actionable risk stratification algorithms.

## Conclusions

We found that AM patients with acute-phase sVA were characterized by older age, intraventricular conduction delay on ECG, and reduced LVF on admission. These clinical parameters were all significantly and independently associated with sVA during hospitalization. These simple clinical parameters, if confirmed by future studies, may assist in decision-making regarding patient placement and the need for continuous ECG monitoring. However, a formal decision-making algorithm, based on the above parameters, could not be recommended at this time until external validation is performed by larger prospective studies.

Declaration of generative AI and AI-assisted technologies in the manuscript preparation process: No generative AI was used in the preparation of this manuscript.

## Supplementary Information

Below is the link to the electronic supplementary material.

Supplementary Material 1

Supplementary Material 2

## Data Availability

No datasets were generated or analysed during the current study.

## Abbreviations

AM: Acute Myocarditis
CMR: Cardiac Magnetic Resonance Imaging
EF: Ejection fraction
EMB: Endomyocardial Biopsy
LBBB: Left Bundle Branch Block
LVF: Left Ventricular Function
NSVT: Non-Sustained Ventricular Tachycardia
RBBB: Right Bundle Branch Block
SCD: Sudden Cardiac Death
sVA: Sustained Ventricular Arrhythmia
TTE: Trans-thoracic Echocardiography
VA: Ventricular Arrhythmia
