# Supplementary material for: Clinical characteristics associated with sustained ventricular arrhythmia among acute myocarditis patients: a multicenter case-control study
Source: J Interv Card Electrophysiol. 2026 Aug 4;69(9):2965–73. doi: 10.1007/s10840-026-02408-5 (PMC13769723; doi:10.1007/s10840-026-02408-5)
Supplement: Supplementary file 1 [file 10840_2026_2408_MOESM1_ESM.pdf]

Supp Figure 1: ROC analysis of multivariable models for sustained VA in acute myocarditis

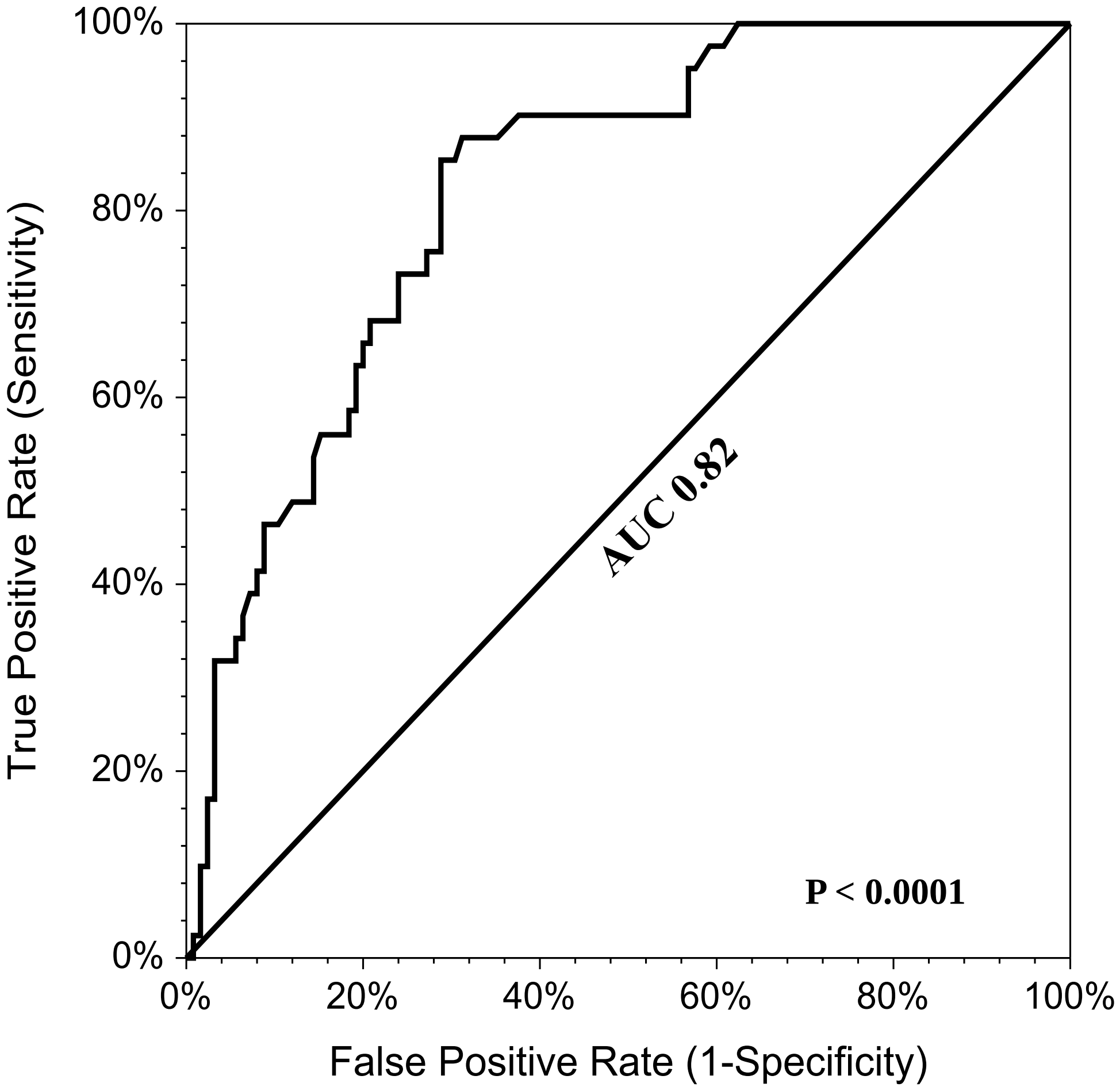

Model 2A

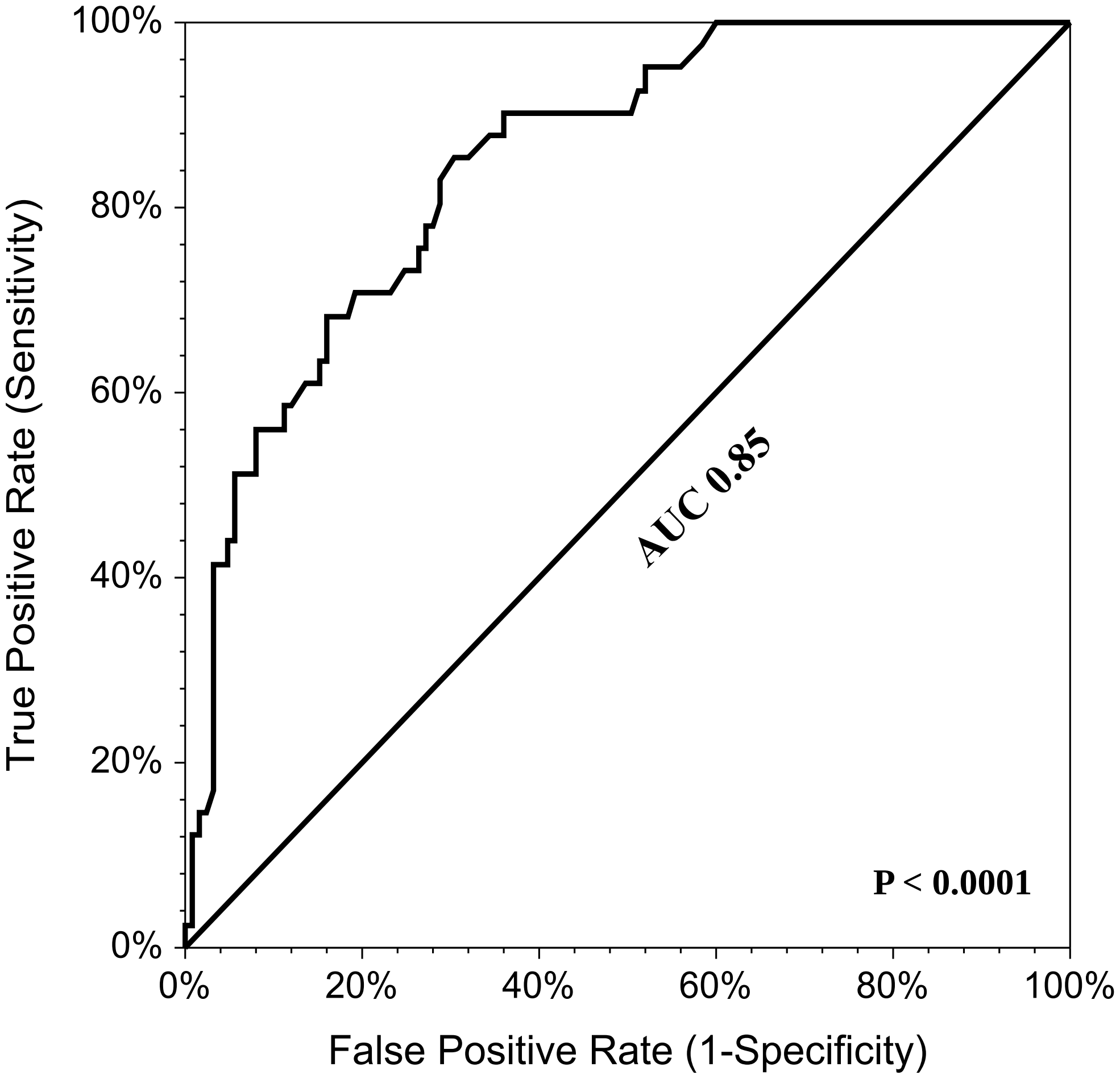

Model 2B
