# Supplementary material for: Clinical characteristics associated with sustained ventricular arrhythmia among acute myocarditis patients: a multicenter case-control study
Source: J Interv Card Electrophysiol. 2026 Aug 4;69(9):2965–73. doi: 10.1007/s10840-026-02408-5 (PMC13769723; doi:10.1007/s10840-026-02408-5)
Supplement: Supplementary file 2 [file 10840_2026_2408_MOESM2_ESM.docx]

Sample size estimation

Our sample size was estimated based on LV function (LVF), which is the most studied prognostic parameter in cardiology and the only clinical parameter whose association with acute-phase VA in AM patients was evaluated (Younis et al, see below). To calculate the sample size needed we estimate reduced LVF (EF<50%) prevalence among AM patients with sVA (‘cases’) as compared with AM patients without VA (‘controls’).

Our estimation of reduced LVF prevalence among AM patients with and without VA is based on Younis et al study (Epidemiology Characteristics and Outcome of Patients with Clinically Diagnosed Acute Myocarditis. Am J Med. 2020; 133: 492-499; ref #24 in our manuscript), which to the best of our knowledge is the only study evaluating the association of LVF on presentation with acute-phase VA in adult AM patients, as mentioned in our discussion (page 14, 1^st^ paragraph). This study evaluated the outcome of 320 AM patients, based on their LVF on presentation. In this study there were 19 patients with in-hospital VA of which 11 had reduced LVF (58%), while there were 301 patients without VA, of which 49 (16%) had reduced LVF. Estimating reduced LVF prevalence of 58% and 16% in AM cases with VA and controls without VA, respectively, the sample size needed for a significance level of 5% and a power of 80% is 11 cases and 44 controls. (calculated via PASS version 26.0.3)

Clinical characteristics associated with sVA among hemodynamically stable AM patients

To evaluate the clinical characteristics associated with sVA among hemodynamically stable AM patients we compared the 20 stable cases to the 128 stable controls in our study (Supp. Table 1). Similar with the results for the whole study population, hemodynamically stable cases were older (median age 50 vs. 36 y/o; p 0.002), presented more often with palpitations (15% vs. 2.3%; p 0.036) and less so with chest pain (30% vs. 77.3%; p<0.001). There were significantly more cases with intraventricular conduction delay (30% vs. 8.8%; p=0.017), and a significantly higher percent of both reduced LVF and moderate-severe/severe LV dysfunction among cases (60% vs. 36.1%; p=0.044, and 30% vs. 10.9%; p=0.04, respectively) (Supp. Table 1).

Supp Table 1: Comparison of patient's characteristics between **hemodynamically stable** cases with sVA and controls without VA during acute phase myocarditis

| Parameter | Sust VA  (n= 20) | No VA  (n= 128) | P |
| --- | --- | --- | --- |
| Age (years) | 50 [40, 56.5] | 36 [25, 48] | **0.002** |
| Female | 7 (35%) | 33 (25.8%) | 0.388 |
| **Medical background** |  |  |  |
| Hypertension | 5 (25%) | 22 (17.2%) | 0.412 |
| Diabetes mellitus | 2 (10%) | 4 (3.1%) | 0.219 |
| Chronic renal failure * | 1 (5%) | 0 (0%) | 0.138 |
| Smoking | 2 (10%) | 25 (19.5%) | 0.327 |
| Alcohol consumption** | 1 (5%) | 15 (11.7%) | 0.412 |
| **Presenting symptoms** |  |  |  |
| Fever | 2 (10%) | 33 (25.8%) | 0.122 |
| Chest pain | 6 (30%) | 99 (77.3%) | **<0.001** |
| Dyspnea | 7 (35%) | 51 (39.8%) | 0.679 |
| Syncope | 3 (15%) | 6 (3.9%) | 0.124 |
| Palpitations | 3 (15%) | 3 (2.3%) | **0.036** |
| Hemodynamic instability | 0 | 0 |  |
| **Lab** |  |  |  |
| WBC (Normal: 4-10^^3^/L) | 10.1 [8.4, 13.8] | 8.9 [7.3, 10.6] | 0.844 |
| CRP (Normal: 0 - 5 mG/L) | 48 [22, 97] | 23 [4.9, 84] | 0.637 |
| Abnormal Troponin admission | 9/12 (75%) | 104/124 (83.8%) | 0.449 |
| CK admission (Normal:40-150 IU/L) | 259 [109, 878] | 342 [131, 583] | 0.692 |
| LDH (Normal: 120-220 IU/L) | 542 [332, 867] | 475 [315,678] | 0.546 |
| **ECG (n=145)** |  |  |  |
| Intraventricular conduction delay  RBBB  LBBB  ICVD | 6/20 (30%)  4/20 (20%)  1/20 (5%)  1/20 (5%) | 11/125 (8.8%)  6/125 (4.8%)  2/125 (1.6%)  3/125 (2.4%) | **0.017**  **0.037**  0.411  0.543 |
| QRS width admission (ms) |  |  |  |
| ST-elevation on admission (n, %) | 5/20 (25%) | 49/125 (39.2%) | 0.233 |
| **Admission TTE (n=139)** |  |  |  |
| Reduced LV function (LVEF< 50%) | 12/20 (60%) | 43/119 (36.1%) | **0.044** |
| Moderate/severe or severe LV dysfunction (LVEF< 35%) | 6/20 (30%) | 13/119 (10.9%) | **0.040** |
| Pericardial effusion on admission | 5/20 (25%) | 16/119 (13.4%) | 0.212 |
| **MRI** (n=125) |  |  |  |
| LGE any | 11/12 (91.6%) | 107/113 (94.7%) | 0.751 |
| LGE anteroseptal | 5/12 (41.7%) | 31/113 (27.4%) | 0.325 |
| LGE inferolateral | 6/12 (50%) | 86/113 (76.2%) | 0.063 |
| Abnormal T1 weighted sequence | 3/12 (25%) | 60/113 (53%) | 0.095 |
| Abnormal T2 weighted sequence | 9/12 (75%) | 102/113 (90.3%) | 0.161 |
